# Interactive Effects of *Nypa fruticans* Fruit Pellets and Dietary Protein Levels on Rumen Fermentation, Gas Kinetics, and Methane Production In Vitro

**DOI:** 10.3390/ani16091313

**Published:** 2026-04-24

**Authors:** Chaturaphat Rueangchuai, Chanon Suntara, Metha Wanapat, Chanadol Supapong, Pongsatorn Gunun, Nirawan Gunun, Suban Foiklang, Payungsuk Intawicha, Anusorn Cherdthong

**Affiliations:** 1 Tropical Feed Resource Research and Development Center (TROFREC), Department of Animal Science, Faculty of Agriculture, Khon Kaen University, Khon Kaen 40002, Thailand; chaturaphat.r@kkumail.com (C.R.); chansun@kku.ac.th (C.S.); metha@kku.ac.th (M.W.); 2 Department of Animal Science, Faculty of Agricultural Innovation and Technology, Rajamangala University of Technology Isan, Nakhon Ratchasima 30000, Thailand; chanadol.su@rmuti.ac.th; 3 Department of Animal Science, Faculty of Natural Resources, Rajamangala University of Technology Isan, Sakon Nakhon Campus, Sakon Nakhon 47160, Thailand; pongsatorn.gu@rmuti.ac.th; 4 Department of Animal Science, Faculty of Technology, Udon Thani Rajabhat University, Udon Thani 41000, Thailand; nirawan.gu@udru.ac.th; 5 Faculty of Animal Science and Technology, Maejo University, Chiang Mai 50290, Thailand; suban@mju.ac.th; 6 Division of Animal Science, School of Agriculture and Natural Resources, University of Phayao, Phayao 56000, Thailand; payungsuk.in@up.ac.th

**Keywords:** Nipa palm, methanogenesis, plant secondary metabolites, rumen ecology, protozoa, feed additive

## Abstract

This study evaluated the effects of *Nypa fruticans* fruit pellet supplementation combined with different CP levels on rumen fermentation characteristics and CH_4_ production using an in vitro gas production technique. A 3 × 4 factorial arrangement was used, consisting of three CP levels (12, 14, and 16%) and four levels of *Nypa fruticans* fruit pellet supplementation (0, 0.5, 1.0, and 1.5% of substrate dry matter), with incubation run included as a random effect in the statistical model. Rumen fluid from Thai native beef cattle was incubated under anaerobic conditions. Gas production kinetics, ruminal pH, ammonia–nitrogen (NH_3_–N), protozoal populations, digestibility, volatile fatty acids (VFA), and CH_4_ production were determined. Significant interactions between CP level and *Nypa fruticans* fruit pellet supplementation were observed for gas production kinetics. Ruminal pH was influenced by CP level at 24 h, while NH_3_–N increased with higher CP levels but decreased with increasing supplementation. Protozoal populations were reduced by *Nypa fruticans* fruit pellets. Methane production was affected by CP level, *Nypa fruticans* fruit pellet supplementation, and their interaction. A clearer reduction was observed at 24 h, particularly at higher supplementation levels. At 24 h of incubation, total VFA, propionate, and butyrate concentrations increased with supplementation, whereas no clear effects were observed at 12 h. In vitro dry matter digestibility was affected at 24 h (*p* < 0.05), but no effect was observed at 48 h, while organic matter digestibility remained unchanged. In conclusion, *Nypa fruticans* fruit pellets, in combination with CP level, modified rumen fermentation patterns and were associated with lower CH_4_ production under in vitro conditions, without negatively affecting digestibility. These findings suggest potential for further in vivo evaluation.

## Simple Summary

Methane (CH_4_) produced during rumen fermentation reduces feed efficiency and contributes to climate change. This study evaluated *Nypa fruticans*, a tropical plant rich in bioactive compounds, as a feed additive using an in vitro system. Different crude protein (CP) levels were combined with *Nypa fruticans* fruit pellet supplementation. The results showed that supplementation was associated with changes in fermentation, including higher propionate and butyrate concentrations and lower protozoal populations. Methane production was reduced, with clearer effects observed at 24 h, particularly at higher CP levels. Digestibility was not negatively affected. Overall, *Nypa fruticans* fruit pellets appear to have potential as a natural feed additive for modifying rumen fermentation and reducing CH_4_ production under in vitro conditions.

## 1. Introduction

Greenhouse gas emissions are a major driver of global climate change [1,2]. Among these gases, methane (CH_4_) from livestock production contributes substantially to agricultural emissions [3,4]. In ruminants, methanogenic archaea produce CH_4_ as a byproduct of rumen fermentation, and the gas is released primarily through eructation [5,6]. Cattle are a major source of CH_4_ emissions in the livestock sector due to their large population and high level of enteric fermentation, while smaller contributions come from buffalo, sheep, goats, and monogastric species [7]. Globally, the ruminant livestock sector is estimated to contribute approximately 30–40% of total anthropogenic CH_4_ emissions, highlighting the need for effective mitigation strategies across production systems. In addition to environmental concerns, CH_4_ production represents an energy loss of approximately 2–12% of gross dietary intake in ruminants [8].

Several strategies have been developed to reduce enteric CH_4_ emissions, including synthetic additives that inhibit methanogenesis. Compounds such as 3-nitrooxypropanol and ionophores can reduce CH_4_ formation; however, their use is often limited by cost, regulatory restrictions, and consumer concerns [9,10,11]. As a result, interest has increased in natural alternatives derived from plant resources.

Plant bioactive compounds are increasingly used as natural feed additives in ruminant diets. These phytogenic additives, derived from herbs, fruits, or plant residues, can improve rumen efficiency and animal performance [12,13,14]. Among them, tannins are polyphenolic compounds known to reduce CH_4_ production by suppressing methanogenic archaea and decreasing ruminal protozoal populations [15,16,17]. Previous studies have shown that rambutan peel powder containing 12% condensed tannins reduced protozoa and CH_4_ production when included at 6% of dietary dry matter (DM) [18]. Similarly, dragon fruit peel powder with 6.90% condensed tannins improved fermentation efficiency and reduced CH_4_ emissions [19]. Tannins can also protect dietary protein from excessive ruminal degradation, thereby improving nitrogen utilization [20]. In addition, mangosteen peel containing condensed tannins and saponins has been reported to enhance microbial protein synthesis and protein utilization [21]. These findings highlight the potential of locally available plant resources as functional feed additives.

Nipa palm fruit (*Nypa fruticans*) is a mangrove species widely distributed across tropical and subtropical coastal regions, particularly in Southeast Asia, where it produces large amounts of underutilized biomass. The fruit develops in large clusters, typically consisting of 50–100 fruits per bunch with an average weight of approximately 7 kg [22]. Despite its high biomass yield, it remains largely underutilized, indicating its potential as a low-cost and locally available feed resource. Previous studies have shown that *Nypa fruticans* fruit contains high levels of phenolic compounds and tannins, which may influence rumen fermentation [22,23]. In addition, pelletization can improve its handling, storage, and feeding properties [24]. Despite these advantages, information on its use as a ruminant feed supplement remains limited.

The interaction between plant secondary compounds and dietary protein supply is also an important factor in rumen fermentation. Adequate nitrogen availability supports microbial growth, and its interaction with bioactive compounds may influence fermentation efficiency and CH_4_ production. However, the combined effects of *Nypa fruticans* and dietary protein level on rumen fermentation, gas production kinetics, and CH_4_ production have not yet been systematically evaluated. Furthermore, dose-dependent effects of *Nypa fruticans* supplementation and its interaction with nitrogen availability remain poorly understood.

Therefore, the objective of this study was to evaluate the effects of *Nypa fruticans* fruit pellets combined with different dietary protein levels on rumen fermentation characteristics and CH_4_ production using an in vitro gas production technique.

## 2. Materials and Methods

### 2.1. Animal Ethics Statement

All procedures were approved by the Institutional Animal Care and Use Committee of Khon Kaen University (IACUC-KKU-17/69) and complied with national animal welfare guidelines.

### 2.2. Preparation of Nypa fruticans Fruit Pellets

*Nypa fruticans* fruit was collected from Nakhon Si Thammarat Province, Thailand, in November 2025. Fruits of uniform size and intact appearance were selected. Only fruits with dark brown to reddish-black coloration were used. The fruits were cut into pieces approximately 4–5 cm long. Samples were sun-dried for 3–4 days to reduce moisture content as commonly applied in plant material processing to preserve bioactive compounds [18,19]. Dried material was ground to pass through a 1 mm sieve (Cyclotech Mill, Tecator, Höganäs, Sweden). Pelletization was performed using a laboratory-scale pellet mill equipped with a 4-mm die. The material moisture content was adjusted to approximately 12–15% before pelleting to improve binding. No external binder was added. During pelletization, the processing temperature was maintained at 60–65 °C to preserve pellet shape and density [24]. The pelleting process was carried out using a Ryuzoukun small pellet machine (Kakiuchi Co., Ltd., Nakajima, Japan). After pelleting, the *Nypa fruticans* fruit pellets were stored in sealed plastic containers at room temperature prior to use.

### 2.3. Animals and Rumen Fluid Collection

Four Thai native beef cattle, approximately 2 years of age and with a body weight of 150 ± 20 kg, were used as rumen fluid donors. The animals were fed a concentrate diet containing 14% CP at 1% of body weight per day, along with roughage, to meet maintenance requirements and ensure stable rumen fermentation conditions. Rice straw was provided ad libitum as the basal roughage. Mineral blocks and clean drinking water were available at all times. The chemical composition of the concentrate diet and rice straw is presented in Table 1. Animals were adapted to the experimental diet for 7 days before rumen fluid collection. On day 7, rumen fluid was collected at 07:00 h prior to morning feeding. Rumen fluid was obtained using a vacuum suction system connected to a flexible stomach tube. The system consisted of a food-grade silicone tube (inner diameter approximately 1.0–1.2 cm) attached to a vacuum pump with adjustable negative pressure. The tube was inserted orally into the rumen to collect representative samples while minimizing salivary contamination. Vacuum pressure was maintained at a moderate level to allow continuous suction without damaging rumen epithelium or excessively foaming the rumen fluid. The first portion of aspirated fluid was discarded to reduce salivary contamination. Approximately 600 mL of rumen fluid was collected from each animal, resulting in a total volume of 2400 mL. The collected rumen fluid was immediately filtered through four layers of cheesecloth and transferred into pre-warmed insulated containers maintained at 39 °C. Samples were transported promptly to the laboratory to preserve microbial activity prior to in vitro incubation.

### 2.4. Experimental Design

The experiment was conducted using a 3 × 4 factorial arrangement. The first factor consisted of three levels of CP in the concentrate diet (12, 14, and 16%). The second factor consisted of four supplementation levels of *Nypa fruticans* fruit pellets (0, 0.5, 1.0, and 1.5% of substrate DM). These inclusion levels were selected based on previous studies involving tannin-rich plant materials, which have shown that relatively low supplementation levels can influence rumen fermentation and CH_4_ production without negatively affecting digestibility [18,19,20]. In particular, supplementation levels in the range of 0.5–2.0% of dietary DM have been commonly applied in in vitro studies evaluating phytochemical-rich feed additives. Therefore, the selected range (0–1.5%) was designed to elicit a measurable biological response while avoiding excessive inclusion levels that may inhibit microbial activity.

*Nypa fruticans* fruit pellets were added as a supplement and were not included in the calculation of dietary CP levels. Each treatment was incubated in five replicate bottles within each incubation run for the determination of gas production kinetics. A total of 60 sample bottles (12 treatments × 5 replicates) and five blank bottles were prepared per run. The experiment was conducted across three independent incubation runs, resulting in 180 sample bottles and 15 blank bottles, for a total of 195 bottles. Individual bottles were considered the experimental units. Incubation run was included as a random effect in the statistical model to account for variation among runs and to minimize potential batch effects.

### 2.5. In Vitro Gas Production Procedure

All feed substrates were ground to pass through a 1-mm screen prior to incubation. Rumen fluid from four donor animals was pooled in equal proportions before mixing with buffer solution. Feed substrates were prepared using 0.5 g of total feed per bottle, with a roughage-to-concentrate ratio of 60:40. Samples were placed into 50 mL serum bottles. Pelletized *Nypa fruticans* fruit was added according to the experimental design. Bottles were flushed with carbon dioxide (CO_2_) to establish anaerobic conditions.

Artificial saliva solution was prepared following a modified method of Menke and Steingass [25]. The solution consisted of 3836.56 mL of distilled water, 2557.71 mL of buffer solution, 1278.85 mL of macro-mineral solution, 0.81 mL of trace mineral solution, 2.27 mL of resazurin solution, and 210.22 mL of reducing solution in a 5000 mL Erlenmeyer flask. The mixture was stirred continuously using a magnetic stirrer and maintained at 39 °C. Oxygen was removed by continuous flushing with CO_2_.

Rumen fluid was filtered through four layers of cheesecloth and mixed with the artificial saliva solution. Forty milliliters of buffered rumen fluid were dispensed into each serum bottle. Bottles were sealed and incubated at 39 °C.

### 2.6. Chemical and Fermentation Analyses

Feed samples were analyzed for DM (method 934.01), ash (method 942.05), and CP (method 984.13; Kjeldahl method) according to AOAC [26]. Neutral detergent fiber (NDF) and acid detergent fiber (ADF) were analyzed according to Van Soest et al. [27] using heat-stable amylase and expressed exclusive of residual ash. All analyses were performed in triplicate, and results were expressed on a DM basis. *Nypa fruticans* fruit was analyzed for total phenolic compounds, total flavonoid compounds, condensed tannins, and saponins. For total phenolic compounds and total flavonoid compounds, dried samples were extracted with 70% ethanol (1:10, *w*/*v*), followed by filtration, and the extracts were used for subsequent analyses. Total phenolic compounds were determined using the Folin–Ciocalteu method at 760 nm, and total flavonoid compounds by the aluminum chloride colorimetric method at 430 nm with slight modifications from Muslykhah et al. [28]. Results were expressed as mg gallic acid equivalents (GAE) and mg quercetin equivalents (QE) per g DM, respectively. Condensed tannins were determined using an acetone-based butanol–HCl assay following Shay et al. [29], with minor modifications. Solvent-soluble, insoluble and total, and water-soluble fractions were extracted using acidified methanol–acetone or distilled water, depending on the fraction. Extracts or reaction mixtures were combined with butanol–HCl and ferric reagent and incubated at 70 °C for 2.5 h for color development. After cooling, absorbance was measured at 550 nm. Condensed tannin concentrations were calculated from a catechin standard curve and expressed as mg catechin equivalents (CE) per g DM. This assay specifically targets condensed tannins (proanthocyanidins), while hydrolyzable tannins were not evaluated. Saponins were determined gravimetrically and expressed as percentage of % DM. All measurements were performed in triplicate, and results are presented as means ± standard deviations. Because each assay targets different compound classes, the values were interpreted independently. Gas production was recorded at 0, 1, 2, 4, 6, 8, 12, 24, 48, 72, and 96 h of incubation. Gas production values were corrected using blank bottles containing buffered rumen fluid without substrate. Net gas production was used for kinetic model estimation. Gas production kinetics were calculated using the model of Schofield [30].

Y = b [1 − exp(−c(t − L))]

where Y is cumulative gas production (mL), b is asymptotic gas volume (mL/g DM), c is the fractional rate constant (h^−1^), and L is the lag time (h). The goodness of fit of the Schofield [30] model was evaluated using the coefficient of determination (R^2^). The R^2^ values ranged from 0.959 to 0.991, indicating an excellent fit of the model to the observed gas production data.

At 12 and 24 h of incubation, gas samples were collected using gas-tight syringes and transferred into 10 mL evacuated glass vials sealed with rubber stoppers and paraffin film. Methane concentration was determined using gas chromatography (Instruments by GC-17A System, Shimadzu; TCD detector; column Shin carbon; column size 3 m × 3 mm, activated charcoal 60/80 mesh, Kyoto, Japan) following the method of Sittijunda et al. [31]. Methane production was expressed as mL CH_4_ per g DM and CH_4_ concentration, determined by gas chromatography. Rumen fluid pH was measured immediately using a pH meter (HANNA HI 98153, Hanna Instruments, Inc., Woonsocket, RI, USA.). 

Liquid samples were divided into two portions. One portion was preserved with 10% formaldehyde at a 1:9 (*v*/*v*) ratio for protozoal enumeration using the direct count method as described by Galyean [32]. Preserved samples were gently mixed prior to counting to ensure uniform distribution. An aliquot of the sample was loaded onto a hemocytometer (Boeco, Hamburg, Germany), and protozoa were counted under a light microscope at 100× magnification without staining. Counts were performed in duplicate and expressed as log_10_ cells/mL. The second portion was acidified with 1 M sulfuric acid at a 1:9 (*v*/*v*) ratio to stop microbial activity and preserve fermentation products. This acidified sample was used for both NH_3_–N determination and volatile fatty acid (VFA) analysis. Ammonia–nitrogen concentration was determined using a spectrophotometric method at 630 nm following Fawcett and Scott [33], with calibration against ammonium sulfate standards. The samples were centrifuged at 10,000× *g* for 15 min, and the supernatant was used for VFA analysis using gas chromatography (Nexis GC-2030, Shimadzu, Kyoto, Japan) equipped with a DB-Wax capillary column (30 m × 0.25 mm × 0.25 μm; Agilent Technologies, Santa Clara, CA, USA). The injector and detector temperatures were set at 200 °C and 250 °C, respectively. Helium was used as the carrier gas at a constant flow rate of 1.0 mL/min. A 1 μL sample was injected with a split ratio of 1:50. The oven temperature was initially set at 100 °C, held for 1 min, and then increased to 180 °C at a rate of 10 °C/min. Individual VFA concentrations were quantified using external calibration standards.

After 24 and 48 h of incubation, fermentation residues were dried overnight at 100 °C. Dry matter was determined according to AOAC [26]. These data were used to calculate in vitro DM digestibility (IVDMD) and in vitro organic matter digestibility (IVOMD). Blank correction was applied using residue from blank bottles. In vitro DM digestibility (IVDMD) was calculated as:

IVDMD (%) = [(initial DM − residual DM)/initial DM] × 100

### 2.7. Statistical Analysis

All data were analyzed using the PROC MIXED procedure of SAS version 9.4 according to a 3 × 4 factorial arrangement. Crude protein level, *Nypa fruticans* fruit pellet supplementation level, and their interaction were included as fixed effects, while incubation run was included as a random effect.

The statistical model used was:

Y_ijk_ = μ + P_i_ + S_j_ + (PS)_ij_ + R_k_ + ε_ijk_

where Y_ijk_ is the observed value, μ is the overall mean, P_i_ is the fixed effect of crude protein level, S_j_ is the fixed effect of *Nypa fruticans* fruit pellet supplementation level, (PS)_ij_ is the interaction effect, R_k_ is the random effect of incubation run, and ε_ijk_ is the residual error. Data were checked for normality and homogeneity of variance using the Shapiro–Wilk and Levene’s tests, respectively. The results indicated that all variables met the assumptions of normality and homogeneity of variance (*p* > 0.05); therefore, no data transformation was required. When significant effects were detected, means were compared using Tukey’s honest significant difference test. Statistical significance was declared at *p* < 0.05.

## 3. Results

### 3.1. Nutrient Content in Experimental Diets

The ingredient and chemical composition of the experimental diets are presented in Table 1. The concentrate diets were formulated to contain 12.0%, 14.0%, and 16.0% CP on a DM basis, and the analyzed CP values corresponded to the formulated levels. Fiber contents (NDF and ADF) were similar among the three concentrate diets. Rice straw contained low CP (2.92% DM) and high structural fiber (72.8% NDF and 55.8% ADF). *Nypa fruticans* fruit contained 1.23% CP and exhibited measurable levels of secondary metabolites, including total phenolic compounds (27.5 ± 1.01 mg GAE/g DM), total flavonoid compounds (19.2 ± 0.88 mg QE/g DM), condensed tannins (137.8 ± 5.04 mg CE/g DM), and saponins (7.6 ± 0.03% DM).

### 3.2. Kinetics of Gas and Cumulative Gas Production

**Figure 1:**
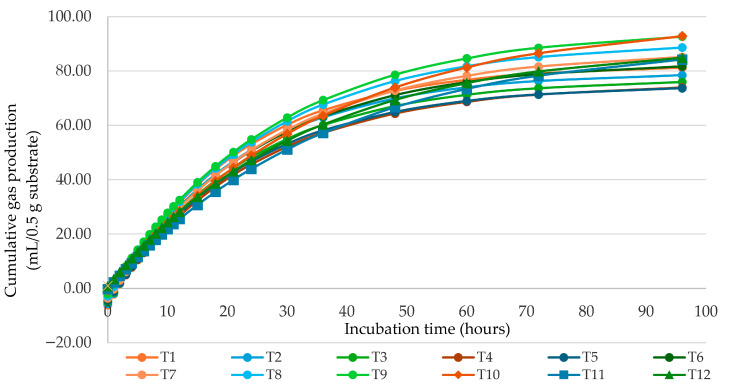
Effect of crude protein level and *Nypa fruticans* supplementation on cumulative gas production after 0–96 h of incubation. T1: 12% CP + 0% *Nypa fruticans* fruit pellet supplementation (Nypa), T2: 12% CP + 0.5% Nypa, T3: 12% CP + 1% Nypa, T4: 12% CP + 1.5% Nypa, T5: 14% CP + 0% Nypa, T6: 14% CP + 0.5% Nypa, T7: 14% CP + 1% Nypa, T8: 14% CP + 1.5% Nypa, T9: 16% CP + 0% Nypa, T10: 16% CP + 0.5% Nypa, T11: 16% CP + 1% Nypa, T12: 16% CP + 1.5% Nypa.

The effects of CP level and *Nypa fruticans* fruit pellet supplementation on gas production kinetics are presented in Table 2. Cumulative gas production during incubation is shown in Figure 1. A significant interaction between CP level and *Nypa fruticans* fruit pellet supplementation was observed for potential gas production (b), fractional rate constant (c), lag time (L), and cumulative gas production at 96 h (*p* < 0.05). At 12% CP, supplementation with *Nypa fruticans* fruit pellets affected potential gas production and lag time. Potential gas production ranged from 77.4 to 112.7 mL/g DM across supplementation levels. Lag time varied from 0.60 to 1.70 h. At 14% CP, gas production remained relatively consistent across treatments. Potential gas production ranged from 119.9 to 126.7 mL/g DM, while lag time ranged from 0.86 to 1.26 h. At 16% CP, the gas kinetics parameters showed moderate variation among supplementation levels. Potential gas production ranged from 82.6 to 89.2 mL/g DM, and lag time ranged from 1.06 to 1.66 h. The pattern of cumulative gas production at 96 h was consistent with the variation observed in potential gas production.

### 3.3. Rumen Parameters and Protozoal Population

The effects of CP level and *Nypa fruticans* fruit pellet supplementation on rumen fermentation characteristics, protozoal populations, and in vitro CH_4_ production are presented in Table 3. For ruminal pH, no interaction between CP level and *Nypa fruticans* fruit pellet supplementation was detected at either 12 or 24 h of incubation (*p* > 0.05). However, CP level influenced ruminal pH at 24 h (*p* < 0.05), whereas *Nypa fruticans* fruit pellet supplementation had no effect at either sampling time. Overall, ruminal pH values remained within a relatively narrow range across treatments.

Ammonia–nitrogen concentration was affected by both CP level and *Nypa fruticans* fruit pellet supplementation at 12 and 24 h of incubation (*p* < 0.05). No interaction between CP level and *Nypa fruticans* fruit pellet supplementation was observed at 12 h or 24 h of incubation (*p* > 0.05). However, increasing CP levels resulted in higher NH_3_–N concentrations, whereas increasing levels of *Nypa fruticans* fruit pellet supplementation led to a decrease in NH_3_–N concentrations.

Protozoal populations were influenced by both CP level and *Nypa fruticans* fruit pellet supplementation at 12 h of incubation (*p* < 0.05), whereas their interaction was not significant. At 24 h, protozoal counts were affected only by *Nypa fruticans* fruit pellet supplementation (*p* < 0.05), whereas CP level and the interaction between factors were not significant (*p* > 0.05).

Methane concentration at both 12 and 24 h was significantly affected by CP level, *Nypa fruticans* fruit pellet supplementation, and their interaction (*p* < 0.01). The presence of a significant interaction indicates that the effect of *Nypa fruticans* supplementation on CH_4_ production varied depending on CP level. At 12 h, CH_4_ concentration was influenced by a linear effect of CP level (*p* = 0.0020), whereas no quadratic effect was observed (*p* > 0.05). In contrast, at 24 h, both linear (*p* = 0.0049) and quadratic (*p* < 0.0001) responses to CP level were significant. Increasing levels of *Nypa fruticans* supplementation reduced CH_4_ concentration, with a strong linear effect at both incubation times (*p* < 0.0001). In addition, quadratic responses were evident at both 12 and 24 h (*p* < 0.01). A cubic effect of *Nypa fruticans* supplementation was detected at 12 h (*p* < 0.0001), but not at 24 h (*p* > 0.05).

### 3.4. In Vitro Digestibility of Dry Matter and Organic Matter

The effects of CP level and *Nypa fruticans* fruit pellet supplementation on in vitro digestibility are presented in Table 4. No interaction between CP level and *Nypa fruticans* fruit pellet supplementation was observed for IVDMD at either 24 or 48 h of incubation (*p* > 0.05). Likewise, CP level alone did not affect IVDMD at either incubation time (*p* > 0.05). However, *Nypa fruticans* fruit pellet supplementation influenced IVDMD at 24 h (*p* = 0.0324), whereas no effect was detected at 48 h (*p* > 0.05). In vitro organic matter digestibility was not affected by CP level, *Nypa fruticans* fruit pellet supplementation, or their interaction at either incubation time (*p* > 0.05).

### 3.5. In Vitro Volatile Fatty Acid

The effects of CP level and *Nypa fruticans* fruit pellet supplementation on in vitro volatile fatty acid (VFA) profiles are presented in Table 5. No interaction between CP level and *Nypa fruticans* fruit pellet supplementation was detected for most VFA parameters, except for total VFA at 12 h and butyrate at 24 h (*p* < 0.05). Crude protein level affected total VFA at both sampling times and influenced acetate, propionate, butyrate, and the C2:C3 ratio at 24 h (*p* < 0.05). *Nypa fruticans* fruit pellet supplementation did not affect VFA profiles at 12 h, but at 24 h it affected total VFA, propionate, butyrate, and the C2:C3 ratio.

## 4. Discussion

### 4.1. Nutrient Content in Experimental Diets

The experimental diets were formulated to provide three levels of CP to represent different nutritional conditions for Thai native beef cattle. The 12% CP diet was below the recommended requirement, whereas the 14% CP diet met the requirement and the 16% CP diet slightly exceeded it. Differences in dietary CP supply can influence rumen microbial activity because microorganisms require adequate nitrogen for microbial protein synthesis and efficient fermentation.

*Nypa fruticans* fruit pellets contained relatively low crude protein but were rich in plant secondary compounds, including tannins, polyphenols, flavonoids, and saponins. These compounds are known to influence rumen microbial activity and fermentation patterns. Polyphenols act as natural antioxidants, while saponins may alter microbial populations and help limit excessive protein degradation [17,34,35]. The levels observed in this study are in line with previous reports using rambutan fruit peel powder, dragon fruit, mangosteen peel powder, and *Wolffia globosa* as dietary additives [18,19,21,29]. The reduction in NH_3_–N observed in this study may reflect reduced protein degradation; however, the specific role of tannins cannot be confirmed because tannin activity was not directly evaluated. The reported tannin concentration is relatively high compared with most tropical feed resources. This may reflect differences in analytical methods or the inclusion of total phenolic fractions rather than condensed tannins alone. Therefore, interpretation of its biological effects should be made with caution. The tannin concentration of *Nypa fruticans* fruit pellets in the present study was higher than that reported for several tropical plant materials, such as rambutan peel and dragon fruit peel powder [18,19]. However, the relatively high tannin concentration may suggest that careful consideration of inclusion level is required, as excessive tannins may inhibit microbial activity and reduce feed utilization. Therefore, the inclusion level of *Nypa fruticans* fruit pellets should be carefully controlled to balance its potential benefits on rumen fermentation against possible inhibitory effects of high tannin concentrations.

### 4.2. Kinetics of Gas and Cumulative Gas Production

The gas production kinetics observed in this study suggest that dietary CP level and *Nypa fruticans* fruit pellet supplementation jointly influenced rumen fermentation activity. The relatively high fermentation potential observed in the 14% CP diet may suggest that this protein level provided sufficient nitrogen for microbial growth and metabolic activity. Adequate nitrogen availability is essential for rumen microorganisms because it supports microbial protein synthesis and promotes efficient degradation of fermentable substrates [36,37].

In contrast, treatments supplemented with *Nypa fruticans* fruit pellets tended to show lower gas production in some cases. This response may be associated with plant secondary compounds; however, these compounds were not directly quantified in this study. The relatively high tannin concentration reported for *Nypa fruticans* in previous studies may suggest that dose-dependent effects should be considered, as excessive tannins have been associated with reduced microbial activity [38]. However, tannin content and its direct effects on fermentation were not determined or isolated in this experiment.

Previous studies have indicated that tannins and other phenolic compounds can interact with microbial enzymes or dietary substrates, potentially reducing fermentation rate [38,39]. In the present study, such mechanisms were not directly evaluated; therefore, their role in explaining the observed gas production patterns remains speculative. Similar responses have been reported for other tannin-containing feed additives, where moderate inclusion levels altered gas production patterns while excessive levels inhibited fermentation [39].

The variation observed among treatments also may suggest that the response to *Nypa fruticans* fruit pellet supplementation depends on the balance between nitrogen supply and the presence of secondary compounds. When dietary protein availability was adequate, moderate supplementation appeared less restrictive to microbial fermentation [36,37]. However, microbial activity and nitrogen utilization were not directly measured in this study, and thus, this interpretation should be considered indirect.

Differences in gas production among treatments may also reflect variation in substrate availability and fermentation dynamics rather than specific biochemical mechanisms. When nitrogen supply was limited, lower gas production was observed in some supplemented treatments; however, the extent to which plant secondary compounds contributed to this response cannot be determined from the present data.

Differences in lag time among treatments may also reflect the influence of plant secondary compounds on early fermentation dynamics. Tannins and phenolic compounds have been reported to affect microbial attachment to feed particles and reduce enzymatic activity during the initial stages of substrate degradation [40]. However, microbial attachment and enzyme activity were not assessed in this study, and therefore this explanation is based on previous reports rather than direct evidence.

### 4.3. Rumen Parameters, Protozoal Population, and In Vitro Methane Concentration

Ruminal pH remained within the range generally considered suitable for normal rumen fermentation. Values within this range support the activity of cellulolytic microorganisms and promote efficient fibre degradation [27]. The absence of a clear effect of *Nypa fruticans* fruit pellet supplementation on ruminal pH may suggest that the inclusion level used in this study did not substantially alter rumen buffering conditions. Similar observations have been reported by De Jong et al. [41], who found that condensed tannin supplementation did not significantly influence ruminal pH under different dietary conditions.

The lower NH_3_–N concentration observed in supplemented treatments may be associated with the phytochemical components present in *Nypa fruticans* fruit. However, the specific contribution of tannins was not directly measured or isolated in this study and therefore cannot be confirmed. The reduction in NH_3_–N observed in this study may reflect reduced protein degradation; however, the contribution of tannins cannot be confirmed because tannin activity was not directly measured [36]. Consistent with this mechanism, previous studies have reported reductions in ruminal ammonia concentration following tannin supplementation [42,43]. Thus, the decrease in NH_3_–N observed here may reflect similar processes, although this remains an indirect interpretation based on the existing literature rather than direct evidence from the present study.

Changes in protozoal populations were observed across treatments. These responses may reflect the influence of plant secondary compounds present in the diet. However, the present experiment did not quantify individual compounds such as tannins or saponins, and their specific roles cannot be distinguished. Protozoal populations decreased with supplementation; however, the specific role of tannins or saponins cannot be distinguished because individual compounds were not quantified [44], while saponins have also been associated with reductions in protozoal populations. In the current study, only protozoal counts were measured; therefore, any link to specific phytochemicals should be interpreted cautiously.

*Nypa fruticans* fruit pellet supplementation reduced CH_4_ production in a time- and diet-dependent manner, particularly at higher CP levels. This response became more evident with increasing incubation time, suggesting that fermentation shifts developed progressively. The lowest CH_4_ value was observed in the diet containing 16% CP with 1.5% supplementation, indicating a potential interaction between nitrogen availability and dietary components. However, the mechanisms underlying this interaction were not directly evaluated in this study.

The reduction in CH_4_ observed in this experiment is consistent with mechanisms reported in previous studies [45,46]. Tannins and saponins have been reported to influence methanogenic activity [47,48], potentially altering hydrogen utilization pathways [49]. However, methanogen abundance, hydrogen balance, and the specific activity of these compounds were not elucidated in the present study, and therefore these mechanisms cannot be confirmed. In addition, although saponins have been reported to reduce protozoal populations [50], saponin content was not quantified here, and thus its contribution remains speculative.

The fermentation shift observed in this study, particularly the increase in C3 and the tendency for a lower C2:C3 ratio, suggests a change in hydrogen utilization. The increase in propionate observed in this study suggests a shift in fermentation pattern; however, hydrogen utilization pathways were not directly measured [50]. The present data support a shift in fermentation pattern; however, hydrogen flow was not directly measured, and this interpretation should be considered indirect.

Previous studies have suggested that tannins and saponins can redirect hydrogen flow away from CH_4_ formation toward alternative pathways such as C3 and C4 production [37,47]. This shift has been proposed to contribute to lower CH_4_ production. In the present study, such mechanisms were not directly assessed, and therefore the observed changes in fermentation profile cannot be attributed to specific compounds.

The present findings are consistent with studies using plant secondary compounds. Ibrahim et al. [16] reported that mimosa tannin reduced CH_4_ production without affecting IVOMD, ammonia, or total VFA at moderate inclusion levels. Similarly, Stypinski et al. [14] demonstrated that several plant secondary metabolites reduced CH_4_ production, although responses varied depending on compound type. In tropical systems, Cruz-Matías et al. [15] showed that plant-based supplementation reduced enteric CH_4_ emissions, supporting their potential application. Adams et al. [12] reported a quadratic response of CH_4_ production to tannin extract supplementation, highlighting the importance of appropriate dosage.

Overall, *Nypa fruticans* fruit pellets appear to influence rumen fermentation patterns under in vitro conditions. However, the underlying mechanisms were not directly measured, and interpretations regarding tannins, saponins, or microbial pathways are based on previously reported evidence rather than direct observations from this study. Future studies should include direct measurements of phytochemical composition, methanogen populations, and hydrogen balance to confirm these mechanisms.

### 4.4. In Vitro Digestibility of Dry Matter and Organic Matter

The absence of changes in digestibility may indicate that the inclusion level of *Nypa fruticans* fruit pellets was insufficient to substantially influence rumen microbial activity under the present in vitro conditions. However, the concentration and activity of specific phytochemical compounds, such as tannins or flavonoids, were not quantified or characterized in this study. Therefore, their direct contribution to digestibility responses cannot be confirmed. Previous studies have suggested that rumen microorganisms can adapt to certain plant secondary compounds over time [51]. However, because microbial adaptation was not directly evaluated in the present experiment, this explanation should be interpreted with caution as an underlying factor here. In contrast, previous studies have reported that higher levels of tannin supplementation can negatively affect nutrient digestibility. Norris et al. [40] demonstrated that increasing inclusion of quebracho tannin (15–45 g/kg DM) resulted in a linear reduction in apparent digestibility, indicating that excessive tannin levels may impair nutrient utilization.

Such negative effects have been attributed in the literature to interactions between plant secondary compounds and rumen microbial processes. For example, tannins and other phytochemicals may interact with microbial enzymes or form complexes with nutrients, which can reduce their availability for digestion [40,52]. However, these mechanisms were not directly assessed in the present study and therefore cannot be used to explain the current results.

Overall, the present findings indicate that *Nypa fruticans* fruit pellet supplementation at the tested levels did not impair in vitro digestibility. The lack of response may suggest that any potential effects of secondary compounds were minimal under these conditions, although their specific roles require further investigation using direct phytochemical and microbial analyses.

### 4.5. In Vitro Volatile Fatty Acid

The effects of CP level and *Nypa fruticans* fruit pellet supplementation on VFA production were more evident at 24 h, while only total VFA changed at 12 h. This pattern may suggest that fermentation responses became more pronounced over time, possibly reflecting increased microbial activity at later incubation stages [53]. Higher CP likely improved nitrogen availability, which supports microbial growth and increases fermentation activity at 24 h. The increase in C3 and C4 with *Nypa fruticans* fruit pellet supplementation indicates a shift in fermentation pathways. Propionate acts as a hydrogen (H_2_) sink by utilizing metabolic hydrogen, thereby reducing its availability for competing pathways [1,4]. In contrast, C2 was not affected, suggesting that the observed shift was primarily associated with increased C3 and C4 production rather than a reduction in C2. The observed changes in VFA profiles may reflect alterations in fermentation patterns; however, hydrogen balance and microbial pathways were not directly measured in this study. Therefore, the mechanisms underlying this shift cannot be confirmed.

Although tannins were not directly measured, previous studies indicate that they can modify microbial activity and hydrogen utilization, thereby influencing VFA profiles [1,6]. In the present study, the concentration and activity of tannins or other phytochemicals were not quantified, and their specific effects cannot be distinguished. Tannins have been reported to influence methanogenic activity in previous studies; however, methanogen populations were not measured, and thus any link between VFA changes and methanogenesis remains indirect. Similarly, although some studies have reported that plant secondary compounds can reduce protozoal populations or alter microbial communities, the present study did not quantify specific compounds such as saponins or evaluate microbial structure. Therefore, these mechanisms should be interpreted cautiously. Previous studies have suggested that plant secondary compounds may influence fermentation pathways, including increasing the proportion of C3 and C4 [14,54]. This response is consistent with Stypinski et al. [14], who reported that several phytochemicals increased C3 and C4 proportions without markedly affecting total VFA. However, in the present study, these responses cannot be attributed to specific compounds, as their presence and activity were not directly measured.

In addition, tannins have been reported to bind to dietary protein, reducing protein degradation and ammonia formation in the rumen [36,37]. While such effects have been described in the literature, protein binding and nitrogen flow were not directly evaluated in this experiment. Therefore, their contribution to the observed VFA profile remains speculative.

The C2:C3 ratio tended to decrease, suggesting a shift in fermentation pathways. However, overall fermentation efficiency and hydrogen partitioning were not directly measured, and thus this interpretation should be considered indirect. Propionate is an important precursor for gluconeogenesis [17], and a higher proportion of C3 is generally associated with improved energy utilization in ruminants [43,55]. These results agree with Wanapat et al. [36] and Polyorach et al. [21], who reported that tannin-rich feeds increased C3 and reduced the C2:C3 ratio. Nevertheless, the present study did not isolate the effects of tannins or other specific phytochemicals, and therefore similar mechanisms cannot be confirmed.

Overall, *Nypa fruticans* fruit pellets appear to modify rumen fermentation patterns under in vitro conditions. However, the underlying mechanisms, including potential roles of tannins, saponins, hydrogen utilization, and microbial interactions, were not directly measured and should be interpreted based on the supporting literature rather than direct evidence from this study.

## 5. Conclusions

Dietary CP level and *Nypa fruticans* fruit pellet supplementation, as well as their interaction, influenced rumen gas kinetics, fermentation characteristics, and CH_4_ production under in vitro conditions. Increasing CP from 12% to 16% enhanced nitrogen availability, which may support microbial activity, while supplementation with *Nypa fruticans* fruit pellets—particularly at 1.5% of DM—was associated with shifts in fermentation patterns, including increased C3 and C4 production and reduced protozoal populations. These responses suggest shifts in hydrogen utilization during fermentation.

The combination of 16% CP with 1.5% *Nypa fruticans* fruit pellets was associated with lower CH_4_ production, without negative effects on in vitro DM or organic matter digestibility. Overall, these findings indicate that *Nypa fruticans* fruit pellets may modify rumen fermentation and reduce CH_4_ production under in vitro conditions. Further in vivo studies are required to confirm these effects and to evaluate their implications for animal performance and practical feeding systems.

## Figures and Tables

**Table 1:** Ingredients and chemical composition of concentrate, rice straw, and *Nypa fruticans* fruit.

Ingredient, % Dry Matter	Crude Protein (CP) Level			Rice Straw	*Nypa fruticans* Fruit
	12%	14%	16%		
Cassava chip	53.3	53.2	52.4	-	-
Soybean meal	9.2	9.3	11.9	-	-
Rice bran	18	18	16.5	-	-
Palm kernel meal	16	16	15.2	-	-
Urea	1	1	1.5	-	-
Premix ^1^	1	1	1	-	-
Sulfur	0.5	0.5	0.5	-	-
Salt	1	1	1	-	-
Chemical composition					
Dry matter, %	89.9	89.9	89.9	90.2	95.9
Crude protein, % DM	12.0	14.0	16.0	2.92	1.23
Neutral detergent fiber, % DM	26.3	26.3	26.2	72.8	84.6
Acid detergent fiber, % DM	13.1	13.1	12.8	55.8	78.8
Phytochemical values ^2^					
Total phenolic compounds, mg GAE/g DM	-	-	-	-	27.5 ± 1.01
Total flavonoid compounds, mg QE/g DM	-	-	-	-	19.2 ± 0.88
Condensed tannins, mg CE/g DM	-	-	-	-	137.8 ± 5.04
Saponins, % DM	-	-	-	-	7.6 ± 0.03

^1^ Premix (1 kg) contains the following vitamins and minerals: vitamin A: 10,000,000 IU; vitamin E 70,000 IU; vitamin D: 1,600,000 IU; Fe: 50 g; Zn: 40 g; Mn: 40 g; Co: 0.1 g; Se: 0.1 g; I: 0.5 g; ^2^ Values are expressed as means ± standard deviations (*n* = 3); DM = dry matter; Phytochemical values are expressed in different units and represent distinct classes of compounds; therefore, they are not additive and should not be summed. Total phenolics, flavonoids, and condensed tannins were determined using different analytical methods and may represent overlapping fractions.

**Table 2:** The effects of crude protein (CP) levels in combination with *Nypa fruticans* fruit pellet supplementation on gas production kinetics parameters and cumulative gas production after 96 h of incubation.

**Crude Protein Level, %**	*Nypa fruticans* Fruit Pellet Supplementation, **% Substrate**	**Kinetics of Gas**			**Cumulative Gas, mL** **/** **g DM**
		b	c	L	
12	0	91.8 ^dc^	0.067 ^a^	1.70 ^a^	89.3 ^c^
	0.5	77.4 ^f^	0.037 ^b^	1.50 ^ab^	77.1 ^d^
	1.0	95.3 ^c^	0.035 ^b^	1.03 ^bcd^	93.4 ^c^
	1.5	112.7 ^b^	0.037 ^b^	0.60 ^d^	112.9 ^b^
14	0	126.7 ^a^	0.034 ^b^	1.26 ^abc^	123.4 ^a^
	0.5	122.4 ^a^	0.035 ^b^	0.86 ^cd^	122.8 ^ab^
	1.0	125.4 ^a^	0.037 ^b^	0.90 ^cd^	125.0 ^a^
	1.5	119.9 ^ab^	0.036 ^b^	0.86 ^cd^	116.6 ^ab^
16	0	87.2 ^cde^	0.040 ^b^	1.53 ^ab^	87.3 ^c^
	0.5	85.9 ^def^	0.041 ^b^	1.66 ^a^	85.3 ^cd^
	1.0	89.2 ^dce^	0.038 ^b^	1.06 ^bcd^	92.5 ^c^
	1.5	82.6 ^ef^	0.044 ^b^	1.13 ^bc^	85.0 ^cd^
SEM		2.863	0.003	0.162	0.6323
Comparison					
Crude protein level		<0.0001	<0.0001	<0.0001	<0.0001
*Nypa fruticans* fruit pellet		<0.0002	<0.0004	<0.0001	<0.0001
CP × *Nypa fruticans* fruit pellet		<0.0001	<0.0008	0.001	<0.0001

Gas production = b [1 − exp(−c(t − L))]; c = a rate constant (per time unit); L = a discontinuous lag term (h); b = the final asymptotic gas volume corresponding to fully digested substrate (mL/g DM). ^a–f^ values in the same column with different superscripts differ (*p* < 0.05); SEM = standard error of the mean.

**Table 3:** Effects of crude protein (CP) level and *Nypa fruticans* fruit pellet supplementation on rumen fermentation characteristics, protozoal population, and in vitro methane (CH_4_) production.

**Crude Protein Level,** %	*Nypa fruticans* Fruit Pellet Supplementation, %	Rumen Parameters							
		pH		**Ammonia–Nitrogen** (**NH_3_–N**)**, mg**/**dL**		**Protozoal Count, log_10_ Cells/mL**		Methane Production, mL CH_4_/g Dry Matter	
		12 h	24 h	12 h	24 h	12 h	24 h	12 h	24 h
12	0	6.76	6.71	12.0	12.9	5.59	5.52	32.8 ^ab^	25.8 ^b^
	0.5	6.70	6.68	11.4	13.0	5.00	5.42	19.7 ^c^	22.8 ^b^
	1.0	6.71	6.76	11.1	12.4	5.10	5.20	15.8 ^c^	21.4 ^b^
	1.5	6.71	6.73	11.4	11.6	5.10	5.20	14.7 ^c^	19.9 ^bc^
14	0	6.70	6.71	13.8	14.4	5.74	5.36	33.6 ^a^	38.1 ^a^
	0.5	6.71	6.68	12.4	14.1	5.4	5.26	15.8 ^c^	36.0 ^a^
	1.0	6.68	6.76	11.8	13.5	5.26	5.36	27.3 ^b^	22.1 ^b^
	1.5	6.75	6.73	10.7	13.4	5.42	5.20	17.7 ^c^	25.3 ^b^
16	0	6.71	6.67	15.9	16.2	5.73	5.43	32.2 ^ab^	32.9 ^a^
	0.5	6.71	6.65	14.0	16.0	5.52	5.20	17.2 ^c^	13.1 ^c^
	1.0	6.65	6.66	12.9	15.5	5.36	5.20	15.9 ^c^	16.7 ^c^
	1.5	6.65	6.71	12.2	15.4	5.36	5.10	15.1 ^c^	11.9 ^c^
SEM		0.075	0.067	0.272	0.216	0.129	0.134	1.010	1.541
Significant									
	CP	0.202	0.008	<0.0001	<0.0001	0.005	0.4185	0.0011	<0.0001
	*Nypa fruticans*	0.41	0.207	<0.0001	0.0001	<0.0001	0.0367	<0.0001	<0.0001
	CP × *Nypa fruticans* fruit pellet	0.321	0.268	0.489	0.615	0.588	0.734	0.0003	0.0003
Main effects									
CP		0.203	0.009	<0.0001	<0.0001	0.001	0.426	0.0011	<0.0001
CP (linear)		0.076	0.019	<0.0001	<0.0001	0.004	0.199	0.0020	0.0049
CP (quadratic)		0.697	0.032	0.074	0.094	0.087	0.871	0.8230	<0.0001
	12	6.72	6.72	11.5	12.5	5.20	5.34	20.8	22.5
	14	6.71	6.72	12.2	13.9	5.46	5.30	23.7	30.4
	16	6.68	6.67	13.7	15.8	5.49	5.23	20.6	18.6
*Nypa fruticans*		0.410	0.219	<0.0001	0.0001	<0.0001	0.037	<0.0001	<0.0001
*Nypa fruticans* (linear)		0.262	0.066	<0.0001	<0.0001	<0.0001	0.006	<0.0001	<0.0001
*Nypa fruticans* (quadratic)		0.276	0.967	0.078	0.598	0.001	0.646	<0.0001	0.0018
*Nypa fruticans* (cubic)		0.541	0.284	0.581	0.370	0.457	0.587	<0.0001	0.7180
	0	6.72	6.70	13.9	14.5	5.69	5.44	32.9	32.3
	0.5	6.71	6.67	12.6	14.3	5.31	5.29	17.6	23.9
	1.0	6.68	6.73	11.9	13.8	5.24	5.25	19.7	20.1
	1.5	6.70	6.72	11.4	13.5	5.29	5.17	15.8	19.0

^a–c^ values in the same column with different superscripts differ (*p* < 0.05); SEM = standard error of the mean.

**Table 4:** Effects of crude protein (CP) level and *Nypa fruticans* fruit pellet supplementation on in vitro dry matter and organic matter digestibility.

**Crude Protein Level,** %	*Nypa fruticans* Fruit Pellet Supplementation, %	In vitro Digestibility			
		**In Vitro Dry Matter Digestibility** (**IVDMD**)**,** %		In Vitro Organic Matter Digestibility (IVOMD), % DM	
		24 h.	48 h.	24 h.	48 h.
12	0	66.9	70.5	80.7	84.3
	0.5	66.3	69.4	80.1	83.3
	1.0	66.7	70.4	80.4	83.2
	1.5	67.1	69.8	80.2	83.1
14	0	68.7	69.8	81.5	84.4
	0.5	65.6	69.6	81.2	83.9
	1.0	66.6	69.9	80.9	83.4
	1.5	66.0	68.8	80.2	82.9
16	0	67.2	70.4	81.7	83.9
	0.5	64.4	70.4	80.7	83.7
	1.0	68.1	68.4	81.7	83.4
	1.5	66.6	69.3	80.6	83.0
SEM		0.631	0.752	0.716	0.677
Significant					
	CP	0.971	0.813	0.698	0.936
	*Nypa fruticans*	0.036	0.844	0.742	0.429
	CP × *Nypa fruticans* fruit pellet	0.311	0.917	0.993	0.965
Main effects					
CP		0.751	0.134	0.081	0.074
CP (linear)		0.626	0.123	0.059	0.051
CP (quadratic)		0.580	0.185	0.221	0.065
	12	66.7	70.0	80.4	83.4
	14	66.7	69.5	81.0	83.7
	16	66.6	69.6	81.2	83.5
*Nypa fruticans*		0.291	0.003	0.714	0.007
*Nypa fruticans* (linear)		0.283	0.017	0.522	0.008
*Nypa fruticans* (quadratic)		0.923	0.006	0.971	0.020
*Nypa fruticans* (cubic)		0.111	0.116	0.337	0.257
	0	67.6	70.2 ^a^	81.3	84.2 ^a^
	0.5	65.4	69.8 ^ab^	80.7	83.6 ^ab^
	1.0	67.1	69.6 ^ab^	81.0	83.3 ^b^
	1.5	66.5	69.3 ^b^	80.4	83.0 ^b^

^a,b^ values in the same column with different superscripts differ (*p* < 0.05); SEM = standard error of the mean.

**Table 5:** Effects of crude protein (CP) level and *Nypa fruticans* fruit pellet supplementation on in vitro volatile fatty acids (VFAs) profiles.

**Crude Protein Level,** %	*Nypa fruticans* Fruit Pellet Supplementation, %	In Vitro Volatile Fatty Acids Profiles									
		Total Volatile Fatty Acid (VFA), mmol/L		Acetate (C2), %		Propionate (C3), %		**Butyrate (C4), %**		**C2 to C3 Ratio**	
		12 h.	24 h.	12 h.	24 h.	12 h.	24 h.	12 h.	24 h.	12 h.	24 h.
12	0	93.3 ^bc^	154.1	60.2	63.4	30.3	28.9	8.05	6.33 ^b^	1.99	2.24
	0.5	110.8 ^ab^	141.0	60.2	61.2	30.0	30.4	7.45	6.91 ^b^	2.04	2.11
	1.0	110.9 ^ab^	138.4	60.5	62.8	30.0	26.8	7.40	7.47 ^ab^	2.02	2.35
	1.5	118.0 ^a^	134.7	60.1	59.8	30.3	35.8	7.41	6.79 ^b^	1.99	1.67
14	0	108.8 ^abc^	122.1	60.9	62.2	29.5	27.1	7.72	8.98 ^a^	2.06	2.30
	0.5	99.1 ^abc^	138.7	60.1	58.1	30.3	27.3	7.38	7.77 ^ab^	1.99	2.13
	1.0	100.0 ^abc^	98.6	59.9	55.6	30.7	36.0	7.35	6.54 ^b^	1.95	1.55
	1.5	99.9 ^abc^	88.7	59.6	55.5	31.0	36.7	7.35	6.45 ^b^	1.92	1.52
16	0	103.2 ^ab^	92.1	59.7	58.4	30.0	34.2	7.44	7.18 ^ab^	1.99	1.71
	0.5	94.4 ^bc^	89.7	60.5	55.4	29.8	35.8	7.42	6.72 ^b^	2.03	1.55
	1.0	91.9 ^bc^	101.2	59.9	55.3	30.3	35.5	7.84	6.38 ^b^	1.98	1.56
	1.5	89.2 ^bc^	72.2	59.8	55.2	30.4	35.6	7.63	6.18 ^b^	1.97	1.55
SEM		1.142	1.7	0.4	0.9	0.4	0.9	0.314	0.361	0.132	0.264
Significant											
	CP	0.001	<0.0001	0.666	0.008	0.756	0.018	0.802	0.024	0.803	0.012
	*Nypa fruticans*	0.969	0.011	0.645	0.117	0.523	0.024	0.346	0.029	0.478	0.050
	CP × *Nypa fruticans* fruit pellet	0.004	0.096	0.554	0.863	0.845	0.072	0.561	0.012	0.776	0.274
Main effects											
CP		0.001	<0.0001	0.658	0.008	0.773	0.019	0.802	0.024	0.823	0.012
CP (linear)		0.002	<0.0001	0.379	0.003	0.901	0.007	0.669	0.365	0.840	0.004
CP (quadratic)		0.823	0.521	0.980	0.427	0.463	0.409	0.620	0.010	0.548	0.788
	12	108.2	142.0 ^a^	60.3	61.8 ^a^	30.2	30.5 ^b^	7.58	6.88	2.01	2.09 ^a^
	14	102.0	112.1 ^b^	60.1	57.8 ^b^	30.4	31.8 ^b^	7.45	7.44	1.98	1.88 ^b^
	16	94.7	88.8 ^c^	60.0	56.1 ^b^	30.1	35.3 ^a^	7.58	6.62	1.99	1.59 ^c^
*Nypa fruticans*		0.955	0.011	0.631	0.117	0.542	0.027	0.348	0.029	0.477	0.050
*Nypa fruticans* (linear)		0.865	0.025	0.256	0.029	0.167	0.004	0.411	0.004	0.141	0.009
*Nypa fruticans* (quadratic)		0.678	0.187	0.577	0.427	0.889	0.376	0.289	0.925	0.776	0.721
*Nypa fruticans* (cubic)		0.826	0.749	0.923	0.542	0.780	0.818	0.220	0.950	0.797	0.730
	0	101.8	122.8 ^a^	60.2	61.3 ^a^	29.9	30.0 ^c^	7.74	7.50	2.01	2.08 ^a^
	0.5	101.4	123.1 ^a^	60.3	58.2 ^b^	30.0	31.1 ^bc^	7.42	7.13	2.02	1.93 ^ab^
	1.0	100.9	112.7 ^ab^	60.1	57.9 ^bc^	30.4	32.7 ^b^	7.53	6.80	1.98	1.82 ^b^
	1.5	102.4	98.6 ^b^	59.8	56.8 ^c^	30.5	36.0 ^a^	7.46	6.47	1.96	1.58 ^c^

^a–c^ values in the same column with different superscripts differ (*p* < 0.05); SEM = standard error of the mean.

## Data Availability

The data supporting the findings of this study are available from the corresponding author upon reasonable request.
